# Influence of Cationic *meso*-Substituted Porphyrins on the Antimicrobial Photodynamic Efficacy and Cell Membrane Interaction in *Escherichia coli*

**DOI:** 10.3390/ijms20010134

**Published:** 2019-01-01

**Authors:** Alexandra N. Hurst, Beth Scarbrough, Roa Saleh, Jessica Hovey, Farideh Ari, Shreya Goyal, Richard J. Chi, Jerry M. Troutman, Juan L. Vivero-Escoto

**Affiliations:** 1 Department of Chemistry, The University of North Carolina at Charlotte, Charlotte, NC 28223, USA; ahurst10@uncc.edu (A.N.H.); bscarbro@uncc.edu (B.S.); roasaleh@email.unc.edu (R.S.); jhovey@wayne.edu (J.H.); fari@uncc.edu (F.A.); 2 The Center for Biomedical Engineering and Science, The University of North Carolina at Charlotte, Charlotte, NC 28223, USA; sgoyal2@uncc.edu (S.G.); rchi1@uncc.edu (R.J.C.); 3 Nanoscale Science Program, Department of Chemistry, The University of North Carolina at Charlotte, Charlotte, NC 28223, USA; 4 Department of Biological Sciences, The University of North Carolina at Charlotte, Charlotte, NC 28223, USA

**Keywords:** photodynamic inactivation, gram-negative bacteria, cationic porphyrin, *E. coli*

## Abstract

Photodynamic inactivation (PDI) is a non-antibiotic option for the treatment of infectious diseases. Although Gram-positive bacteria have been shown to be highly susceptible to PDI, the inactivation of Gram-negative bacteria has been more challenging due to the impermeability properties of the outer membrane. In the present study, a series of photosensitizers which contain one to four positive charges (**1**–**4**) were used to evaluate the charge influence on the PDI of a Gram-negative bacteria, *Escherichia coli* (*E. coli*), and their interaction with the cell membrane. The dose-response PDI results confirm the relevance of the number of positive charges on the porphyrin molecule in the PDI of *E. coli*. The difference between the Hill coefficients of cationic porphyrins with **1**–**3** positive charges and the tetra-cationic porphyrin (**4**) revealed potential variations in their mechanism of inactivation. Fluorescent live-cell microscopy studies showed that cationic porphyrins with **1**–**3** positive charges bind to the cell membrane of *E. coli*, but are not internalized. On the contrary, the tetra-cationic porphyrin (**4**) permeates through the membrane of the cells. The contrast in the interaction of cationic porphyrins with *E. coli* confirmed that they followed different mechanisms of inactivation. This work helps to have a better understanding of the structure-activity relationship in the efficiency of the PDI process of cationic porphyrins against Gram-negative bacteria.

## 1. Introduction

Since the discovery of antibiotics, treatment of bacterial infections has become increasingly difficult due to antibiotic resistance. The presence of antibiotic resistant bacteria has led to an increase in infections, healthcare costs and deaths throughout the world [1]. Therefore, it is critical to develop efficient alternatives to kill pathogens. Photodynamic inactivation of microbes (PDI) is a non-antibiotic alternative treatment that uses light, a photosensitizer (PS), and molecular oxygen to create reactive oxygen species (ROS) such as singlet oxygen (^1^O_2_), superoxide and hydroxyl radicals [2,3,4,5,6,7,8,9]. In PDI, the type II mechanism of photochemical reactions, which is associated with the generation of ^1^O_2_, is usually the major pathway in cellular oxidative damage of bacteria [10,11]. ROS oxidize biomolecules such as DNA, proteins and lipids, causing oxidative cellular damage, leading to cell death [12,13].

Gram-positive bacteria are more susceptible to PDI than Gram-negative due to the lack of a complex outer membrane [4]. The complexity of the outer membrane in Gram-negative bacteria creates an impermeable barrier to a PS [2,3,12,13]. For several decades, porphyrin derivatives with positive charges on the porphyrin macrocycle have been used to increase the photosensitivity of Gram-negative bacteria by improving the interaction between the PS and the bacterial cell membrane through electrostatic interactions [4,7,13,14,15,16,17]. The number of charges and their distribution in the porphyrin macrocycle have an important role on PDI efficiency to treat Gram-negative bacteria [18,19,20,21,22,23]. The PDI of cationic porphyrins against *E. coli* increases with the number of charges, with porphyrins containing three or four positive charges being the most efficient [18,23]. Although it is consensual that the increase in the charge number in the porphyrin are highly relevant in the PDI process, several authors have reported that the tri-cationic are more effective than the tetra-cationic derivatives [18,21,23,24]. It has been suggested that the deviation from the predicted trend is due to the amphiphilic properties of the tri-cationic derivatives [23]. However, other factors such as the cell membrane interaction and internalization can also play a role to account for those results.

**Figure 1:**
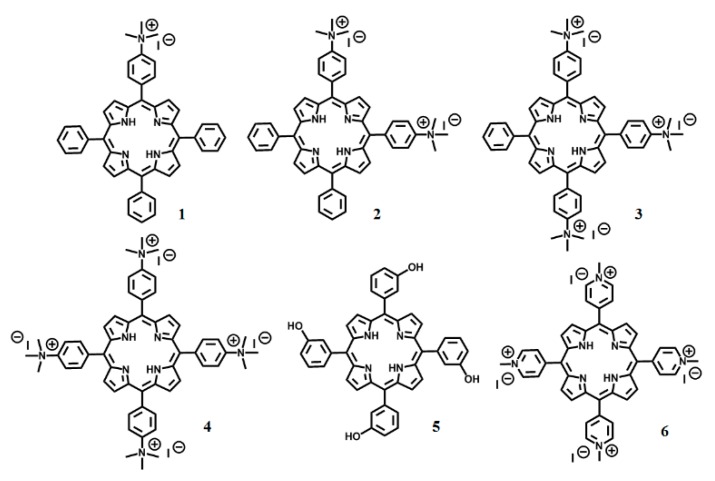
Chemical structure of the porphyrins used in this work. Four cationic porphyrin derivatives were synthesized: **1**–**4** with one to four positive charges, respectively. Compound **2** is a mixture of the *cis* and *trans* isomer. **5** and **6** are commercially available and used as control compounds.

In this work, we studied the PDI and the interaction of four cationic *meso*-substituted porphyrin derivatives with Gram-negative bacteria using *Escherichia coli* (*E. coli*) as a model organism (Figure 1). The PDI effect was studied using the drop plate technique [25]. The PDI efficiency of the **1**–**4** porphyrins against *E. coli* followed the order: **1** < **2** < **4**~**3**. The Hill coefficients for compounds **1**–**3**, obtained from the dose-response graphs, are different than porphyrin 4 as an indication that they may have a different mechanism of inactivation. By using fluorescent live-cell microscopy, we found that the tri-cationic porphyrin (**3**) binds to the membrane of the cell, but the tetra-cationic porphyrin derivative (**4**) is internalized by *E. coli*. The different mechanism of interaction between **3** and **4** with *E. coli* can explain the PDI outcome. Understanding the mechanism of interaction between bacterial cells and PSs is key for advancing PDI as an anti-microbial alternative.

## 2. Results

### 2.1. Syntheses and Structural Characterization of Cationic Porphyrins (***1***–***4***)

To test the influence of charge on the effectiveness of cationic porphyrins, we synthesized a series of cationic porphyrins (**1**–**4**) (Figure 1). The cationic trimethyl ammonium phenyl porphyrin derivatives were synthesized via methylation of amino phenyl-substituted porphyrins. The mono- and tetra-amino phenyl porphyrin derivatives are commercially available. However, the di-mixture and tri-amino phenyl-substituted porphyrins were obtained in a two-step synthetic approach (Scheme S1). First, the nitration of the para position of the tetraphenyl porphyrin (TPP) was carried out using NO_2_BF_4_ as a nitrating agent [26]. The resulting nitro groups were then reduced with SnCl_2_ in acidic medium [27]. In the final step, the amino phenyl porphyrin derivatives were alkylated using a large excess of methyl iodide [28]. The structural properties for the final molecules were confirmed by FT-IR, ^1^H NMR, and ESI-MS or MALDI-TOF MS.

### 2.2. Spectroscopic Characterization

**Figure 2:**
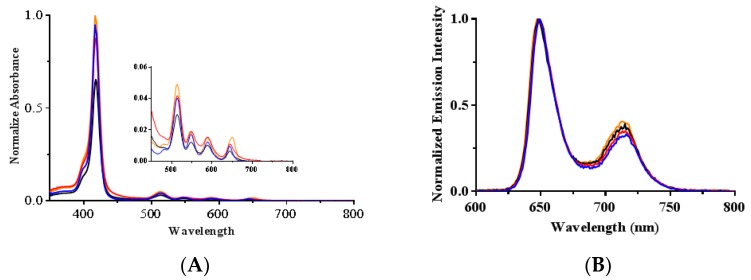
(**A**) Normalized absorption and (**B**) emission spectra for 10 µM solutions of **1** (blue), **2** (red), **3** (black) and **4** (orange) in DMSO. The four Q absorption bands are shown in the inset.

The cationic porphyrins were characterized by UV-vis and fluorescence spectroscopy. Normalized absorption spectra of the cationic porphyrins (**1**–**4**) solutions in dimethyl sulfoxide (DMSO) showed the typical Soret and Q-bands for porphyrins around 415, 515, 550, 590 and 650 nm (Figure 2A). The Soret band wavelengths and the corresponding extinction coefficient values are presented in Table 1. The steady-state fluorescence emission spectra with normalized intensities showed two characteristic emission peaks for free-base porphyrins at 650 and 715 nm (Figure 2B). The emission wavelengths are provided in Table 1.

Fluorescence quantum yields (Φ_F_) in DMSO were calculated relative to tetraphenylporphyrin (TPP) in benzene. The data show that the **1**–**4** cationic porphyrin derivatives have similar fluorescence quantum yield values to the reference compound TPP (Table 1).

**Figure 3:**
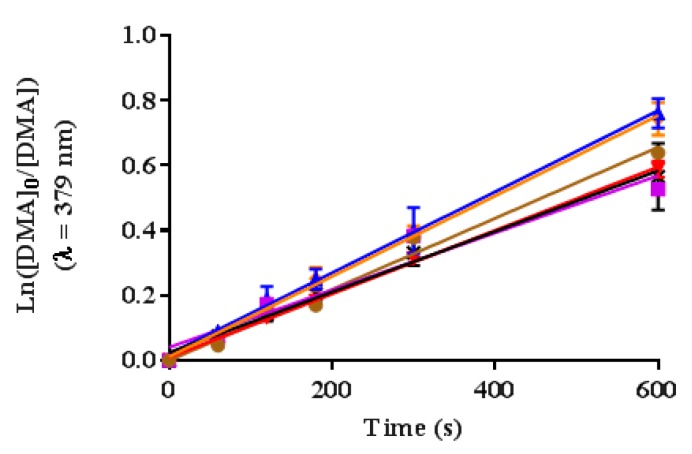
Time-dependent plots for the decay of DMA photosensitized by **1** (blue), **2** (red), **3** (black), **4** (orange), **5** (purple) and **TPP** (brown) in DMF at irradiation wavelength of 515 nm. Values include the mean and standard deviation of three independent experiments.

The ^1^O_2_ generation of the cationic porphyrin derivatives in dimethyl formamide (DMF) was detected using 9,10-dimethylanthracene (DMA). This probe reacts with ^1^O_2_, undergoing a 1,4-cycloaddition that is detected as a decrease in the intensity of the DMA absorption band at 379 nm [29]. The ^1^O_2_ quantum yield was calculated relative to the reference TPP (Φ_Δ_ = 0.62) [21] using the slope of the time-dependent decomposition of DMA plots (Ln([DMA_0_]/[DMA]) versus irradiation times) and Equation (1) [21,29]. The experimental design was confirmed by comparing two porphyrins with known ^1^O_2_ quantum yields in DMF, TPP (Φ_Δ_ = 0.62) [21] and **5** (Φ_Δ_ = 0.54) [29]. The measured quantum yield values matched the literature values within ±2% error (Φ_Δ_ for TPP = 0.62 and Φ_Δ_ for **5** = 0.55) (Figure 3 and Table 1).

(1) $$\Phi_{\Delta ,S}=\;\Phi_{\Delta ,R}\;\times \;\frac{m_{S}}{m_{R}}\;\times \;\frac{1\;-\;10^{-Abs_{R}}}{1\;-\;10^{-Abs_{S}}}$$

The ^1^O_2_ quantum yields were calculated as 0.65 ± 0.04, 0.61 ± 0.03, 0.61 ± 0.06, and 0.65 ± 0.04 for **1**, **2**, **3** and **4**, respectively. Similar to the Φ_F_ results, the Φ_Δ_ were not affected by the amount of cationic substituents.

### 2.3. Photodynamic Inactivation of E. coli

**Figure 4:**
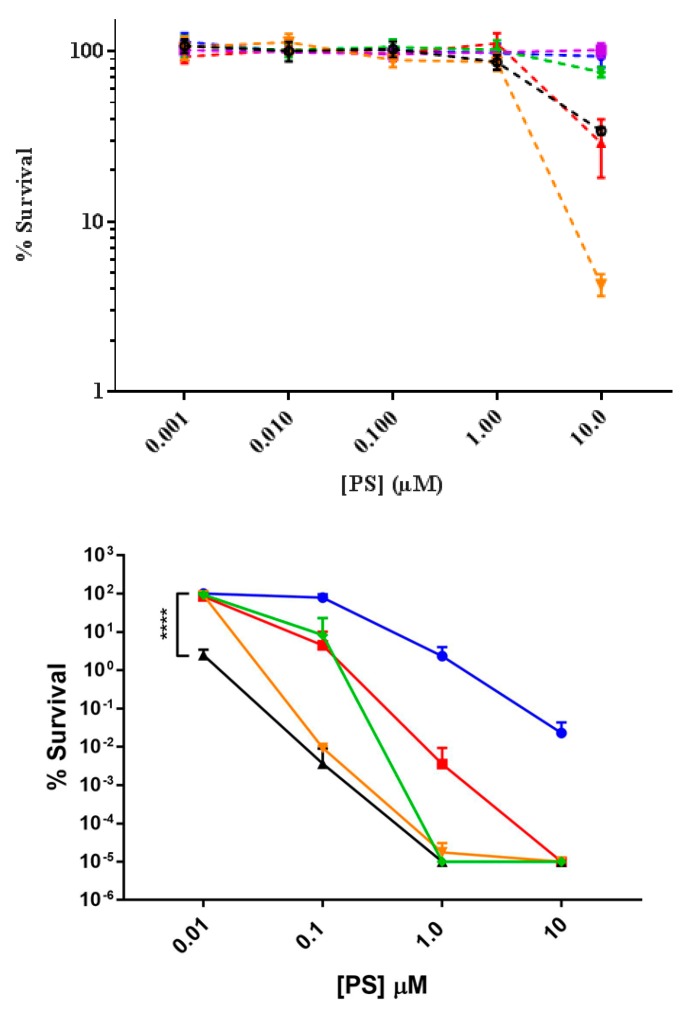
Bacterial toxicity with **1** (blue), **2** (red), **3** (black), **4** (orange), **5** (purple) and **6** (green) against *E. coli* in the absence of light (top) and after light exposure for 20 min (bottom). *E. coli* was incubated with cationic porphyrin derivatives for 30 min. Minimal dark toxicity was observed at concentrations below 1 µM. Values represent the average of three independent experiments. Error bars represent one standard of deviation. Statistical analysis was performed by one-way ANOVA (**** *p* < 0.0001).

The dark toxicity of the cationic porphyrin derivatives were tested against *E. coli* using the drop plate technique with concentrations between 0.001 and 10 µM after 30 min of incubation [25]. The effect of the PS was evaluated based on the number of viable CFU per mL in comparison to a control that was not incubated with a PS (Figure 4). The dark toxicity effect of the synthesized **1**–**4** porphyrins was also compared to a commercially available tetracationic porphyrin derivative commonly used in PDI (**6**), and a neutral porphyrin derivative (**5**) [18,22,30,31]. The cationic porphyrin derivatives showed minimum dark toxicity to *E. coli* at concentrations of ≤1 µM (Figure 4). At 10 µM a reduction of survival of ~0.5–1.5 log units was observed for compounds **2**, **3** and **4**; nevertheless, porphyrins **1**, **5** and **6** showed minimum dark toxicity to *E. coli*. The PDI efficiency of compounds **1**–**6** at concentrations 0.01, 0.1, 1.0 and 10 µM was tested after exposure of treated cultures to light. At 0.01 µM only compound **3** affected the viability of the cells decreasing the number of CFU by 2 log units (*p* < 0.0001). However, at 0.1 µM compounds **3** and **4** were equally effective with a reduction in the viability by 4 log units. Both compounds **3** and **4** were significantly more potent than the other PS at 0.1 µM. At 1.0 µM compounds **3**, **4**, and **6** decreased viability to the limits of detection (7 log units) of our assay (*p* < 0.0001). All but compounds **1** and **5** decreased viability to the detection limit at 10 µM, and compound **1** decreased viability to the limit of detection at 100 µM (data not shown). The neutral porphyrin **5** completely failed to inactivate *E. coli* even at 100 µM (data not shown).

The ability of compound **3** to increase in relative potency to compound **4** prompted us to investigate the PDI efficiency more precisely using an EC_50_ and Hill coefficient analysis with concentrations ranging from 0.001 to 100 µM (Figure S1). Concentrations were chosen at each order of magnitude within the range then focused around the apparent EC_50_ value to improve measurement of the Hill coefficient. Based on the EC_50_ (Table 2) the relative potency of the compounds followed the order: **5** < **1** < **6** < **2**~**4** < **3**. While it was not obvious from EC_50_ plots, further inspection of the log of the percent survival relative to concentration of PS indicated that there was a significant difference between the slopes of the plots for compounds **4** and **6** relative to **1**–**3** (Figure S1). To best fit the data Hill coefficients were assigned as 2.5 for compounds **1**–**3**, 6 for compound **4**, and 4 for compound **6**. These coefficients were based on best fit analysis of the log plots for each, and was weighted towards the higher concentrations that did not result in complete inactivation. Changes in the Hill coefficient had little influence on the measured EC_50_ values. However, the Hill coefficients did greatly influence values for the more commonly used antimicrobial value EC_99.99_. Interestingly, the EC_99.99_ values order of potency was **5** < **1** < **2** < **6** < **4**~**3** (Table 2). With the exception of the tetra-cationic porphyrins there was a clear increase in potency with increase in charge whether we used EC_50_ or EC_99.99_ as the potency parameter. As noted in other works, the tetra-cationic porphyrins were either less potent or equally potent to the tri-cationic porphyrins [21,23,24]. The unusual influence of the fourth charged group and the difference in the Hill fitting parameter suggested that the cationic porphyrins may have an alternative mechanism of activity relative to the mono, di, and tri cationic compounds **1**–**3** [32].

### 2.4. Investigation of Porphyrins ***3*** and ***4*** Interactions with E. coli Using Fluorescent Live-Cell Microscopy

**Figure 5:**
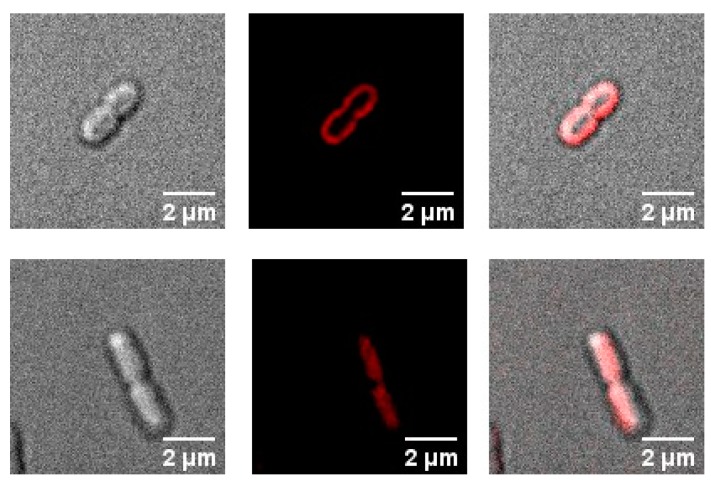
Micrographs depicting the interaction of cationic porphyrins with *E. coli*. **3** (top row) and **4** (bottom row). Left: Differential interference contrast image, middle: fluorescence image (medial z slice), and right: merge image. [PS] = 1 µM; Incubation time: 30 min.

In this work, we used fluorescent live-cell microscopy to directly visualize the binding of PS to *E. coli*. Two different concentrations of porphyrin compounds (**1**–**4**, **6**) were tested, 0.1 and 1 µM; however, 0.1 µM was too low for the fluorescence microscope to detect any signal from those samples (data not shown). Compounds **1**–**3** are localized at the membrane of the *E. coli* cells (Figure 5 and Figure S2) after incubation of 1 µM for 30 min in the dark. The tetra-cationic porphyrins **4** and **6** appeared to be localized intracellularly (Figure 5 and Figure S2).

### 2.5. Study of the Internalization Time-Dependence of Porphyrin ***4*** with E. coli Using Fluorescent Live-Cell Microscopy

**Figure 6:**
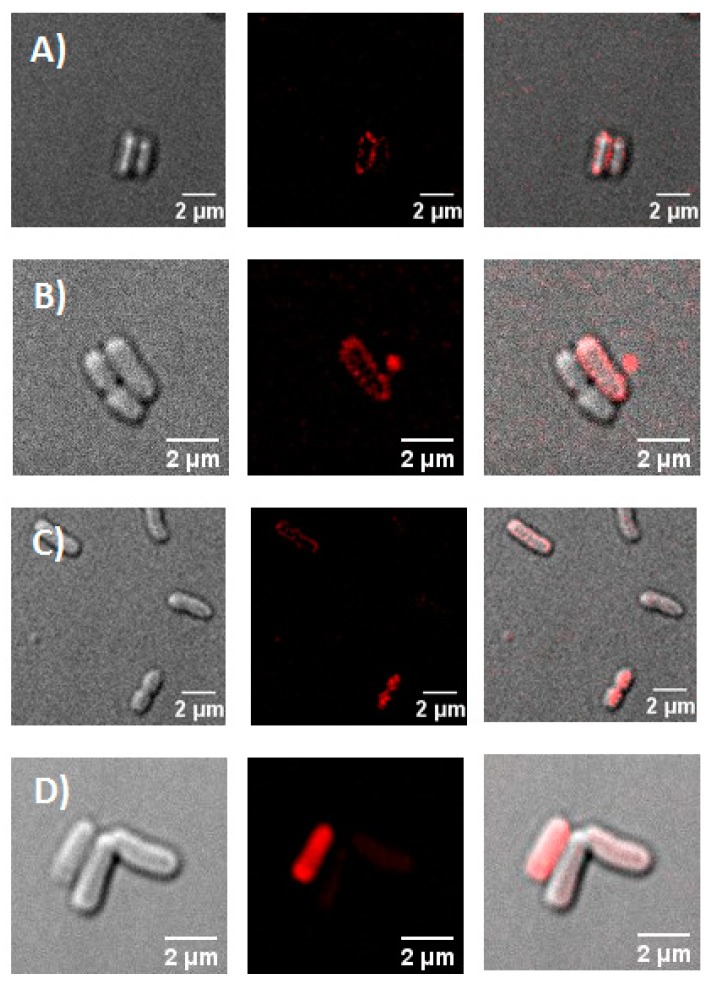
Micrographs of cationic porphyrin **4** with *E. coli* at various incubation times. (**A**) 5, (**B**) 10, (**C**) 15, and (**D**) 20 min. Left: Differential interference contrast image, middle: fluorescence image (medial z slice), and right: merge image. [PS] = 1 µM.

To understand if the uptake of the cationic porphyrins was time dependent, additional microscopy experiments were conducted. *E. coli* cells were incubated in the dark with porphyrin **4** for the following times: 5, 10, 15 and 20 min (Figure 6). After 5 and 10 min, **4** was localized at the membrane of *E. coli* cells. However; after 15 min, porphyrin **4** was observed extracellularly and intracellularly, and after 20 min, the porphyrin was found localized primarily inside the bacterial cells.

### 2.6. Study of the Internalization of Porphyrins ***3*** and ***4*** in E. coli through the Self-Promoted Uptake Pathway

**Figure 7:**
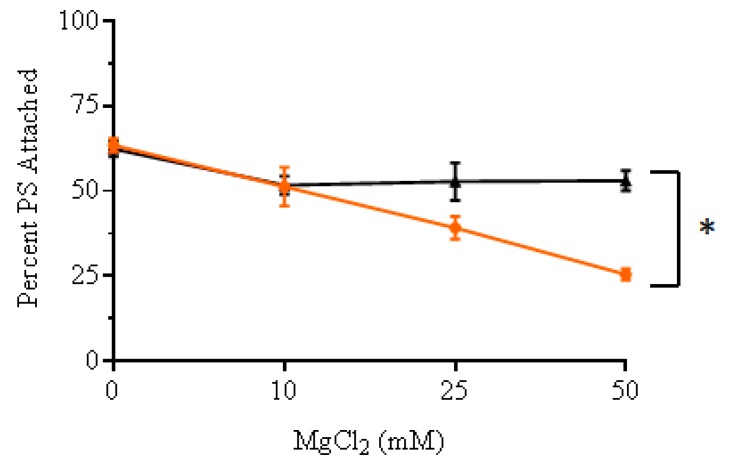
Trend of cellular interaction between cationic porphyrins **3** (black) and **4** (orange) and *E. coli* cells as a function of increasing concentration of Mg^+2^ ions, source MgCl_2_ (0, 10, 25, 50 mM). The percentage of PS attached was calculated as a percentage of the original PS concentration (1 µM). Values represent the average of three independent experiments. Error bars represent one standard of deviation. Statistical analysis was performed by one-way ANOVA (* *p* < 0.05).

To confirm that the cellular internalization of cationic porphyrin **4** in bacterial cells occurs via the self-promoted uptake pathway, the uptake of **4** into *E. coli* cells was studied by incubating the porphyrin in culture solutions pre-exposed to increasing concentrations of Mg^+2^ (Figure 7). The ability of Mg^+2^ to inhibit the uptake of porphyrin **4** was compared against **3**. The concentrations of Mg^+2^ was varied between 0 and 50 mM while the concentration of cationic porphyrins was kept constant. The amount of cationic porphyrin associated with *E. coli* cells was determined by measuring fluorescence of the PS in cell lysates obtained by treatment with 2% sodium dodecyl sulfate (SDS). Calibration curves were constructed for each PS in 2% SDS (Figure S3). Increasing the concentrations of Mg^+2^ caused a higher reduction in the amount of **4** associated to *E. coli* compared to **3**. Incubating cells with 50 mM Mg^+2^ reduced the uptake of tetra-cationic porphyrin **4** by 35% in comparison to only 10% for the tri-cationic compound **3** (*p* < 0.05).

## 3. Discussion

In the present study, a series of photosensitizers which contain one to four positively-charged groups were successfully synthesized and characterized (Figure 1 and Figure 2, and Table 1). The fluorescence quantum yields (Φ_F_) were determined to indirectly characterize the efficiency with which the cationic porphyrin derivatives undergo intersystem crossing (ISC) to the excited triplet state, an essential step in ROS generation [33]. Porphyrin derivatives typically generate low Φ_F_ indicating that the majority of photons absorbed by porphyrins undergo ISC to the excited triplet. The data show that the **1**–**4** cationic porphyrin derivatives have similar Φ_F_ values to the compound reference TPP (Table 1). These results demonstrate that the addition of trimethylammonium groups at the peripheral substituents did not alter the photophysical properties of the cationic porphyrins.

The ^1^O_2_ quantum yields (Φ_Δ_) give an indication of the potential of the cationic porphyrin derivatives for PDI (Figure 3 and Table 1). The Φ_Δ_ were calculated as 0.65 ± 0.04, 0.61 ± 0.03, 0.61 ± 0.06, and 0.65 ± 0.04 for **1**, **2**, **3** and **4** in DMF, respectively [20,21]. Similar to the fluorescence quantum yield results, the Φ_Δ_ were not affected by the cationic substituents. Nevertheless, in this work despite that cationic porphyrins **1**–**4** have statistically similar Φ_Δ_, the PDI performance is quite different. It has been extensively confirmed that the generation of ^1^O_2_ is essential for the PDI process [34,35]; however, it is not the only factor that determines the overall PDI efficiency [23]. In particular, the interaction of the PS with the cell membrane plays a major role. It is well-established that due to the ^1^O_2_ short lifetime, the PS must be close enough to the bacterial membrane or even inside the cell, to cause efficient cell damage [36].

The cationic porphyrin derivatives showed minimum dark toxicity to *E. coli* at concentrations ≤1 µM (Figure 4). At 10 µM the survival percentage was reduced to ~0.5, 0.5 and 1.5 log units for **2**, **3** and **4**, respectively. The reason for the dark toxicity can be explained by the mechanism of interaction between the cationic PS and *E. coli*. The positive charges on the PS molecules promote electrostatic interactions with negatively-charged lipopolysaccharides (LPS) molecules, displacing divalent cations and creating pores in the outer membrane via the self-promoted uptake pathway [15]. At higher concentrations the damage done to the outer membrane may result in lysis of the cells leading to the observed dark toxicity.

The dose-response graph showed that the PDI efficiency of the porphyrins against *E. coli* after 30 min of incubation and concentrations between 0.01 to 1.0 µM was directly correlated with the number of positive charges up to the tri-cationic compound following the order: **5** < **1** < **2** < **3** (Figure 4). However, this trend did not hold for the tetra-cationic compounds **4** and **6**. Both cationic porphyrin derivatives had an increased Hill slope relative to the other porphyrins tested, which led to an uneven increase in potency with increased concentration relative to the other PS. This effect has been previously described for cationic peptides, leading to the conclusion that two activity parameters should be taken into account when considering the potency of an antimicrobial compound [32]. These parameters are the EC_50_ and the activity slope, a parameter related to the Hill coefficient. In particular, the Hill slope may indicate alternative mechanisms of inactivation for the antimicrobial compounds. The contrast in Hill coefficients observed in this work for compounds **4** and **6** compared to porphyrins **1**–**3** may indicate that these compounds interact differently with *E. coli* cells resulting in variations in the PDI effect.

The least effective PS against *E. coli* was the neutral porphyrin derivative **5**, which failed to reduce cell survival even at the highest concentration tested. The lack of PDI effect is likely due to its low binding to the negatively charged surface of *E. coli* cells. These results are consistent with previous literature reports of neutral porphyrin derivatives [4,30]. Porphyrin **1** did not reduce the *E coli* to the detection limit until the concentration of 100 µM was reached, with a 4.0 log reduction at 10 µM. This result has been associated with its aggregation in cell media due to poor water solubility, which leads to low ^1^O_2_ generation [23]. The di-cationic porphyrin derivative **2** in this work is a mixture of the *cis* and *trans* isomers, therefore it is not possible to evaluate the individual effect of each isomer. Nevertheless, the effect of the distribution of charges on the PDI efficiency has already been studied [23,37]. Kessel et al. showed that the *cis* isomer is more efficient than the *trans* isomer due to distortion of the macrocycle induced by the electrostatic repulsion between the neighboring charged groups [37]; and Almeida reported that some possible aggregation of the *trans* isomer can also affect its PDI performance [23]. 

This study shows that the tetra- (**4** and **6**) and tri-cationic (**3**) porphyrins are the most efficient PSs reaching complete inactivation of *E. coli* cells to the detection limit (~7.0 log reduction) in the range of 1–10 µM. Similar PDI performance for tri- and tetra-cationic porphyrins have been reported by other groups [18,21,23,24,38]. Almeida et al. reported a comprehensive study to understand the structure-activity relationship of cationic porphyrins in the efficiency of the PDI process [23]. Three main factors accounted for the difference in PDI effect of the cationic porphyrins; the amphiphilic character of the molecule, the generation of ^1^O_2_ and the affinity to the bacterial cells. In particular, the higher PDI efficiency of the tri- versus the tetra-cationic porphyrins was associated with the amphiphilic character of the tri-cationic molecule, which enhances its affinity for bacteria.

Another factor that is important to consider for the PDI process is the actual localization of the porphyrin in bacteria [36,38,39]. Localization of PSs in prokaryotic cells has been previously demonstrated using fluorescence microscopy [40]. In this study, we observe by fluorescence live-cell microscopy different localizations between porphyrins **1**–**3**, and **4** and **6** with *E. coli* (Figure 5 and Figure S2). At 30 min of incubation, compounds **1**–**3** are localized outside of the *E. coli* membrane; however, porphyrins **4** and **6** have been internalized by the cells. The interaction of **4** with *E. coli* cells is time-dependent, at short times of incubation (<15 min) the porphyrin **4** is localized outside of the membrane (Figure 6). Ragàs et al. used a combination of spectroscopic and time-resolved photophysical techniques to indirectly study the interaction of 5,10,15,20-tetrakis(N-methylpyridinium)porphyrin iodide with *E. coli* cells. Their results showed that the PS was bound to both the outer membrane of the cell and internalized in cells [36].

Fluorescent live-cell microscopy studies revealed the internalization of cationic porphyrin **4**, which may occur via the self-promoted uptake pathway [41]. This pathway associates the cell uptake of cationic PSs with their binding to LPS resulting in displacement of divalent cations and the development of gaps in the outer membrane. The presence of large cationic PS widens the pores in the LPS layer allowing uptake of molecules inside the cell. The internalization of **4** into the cytoplasm of the bacterial cell may explain why these porphyrins produced a different PDI effect than **3**. This observation is also supported by the Hill coefficients in Table 2. It has been shown that there are two main molecular targets, which has been proposed for the photodamage caused to bacteria by PDI. The external structures such as cytoplasmic membrane and cell walls, and internal targets like respiratory complexes, metabolic enzymes and nucleic acids [35,36,38,42,43]. In this study, our results showed that cationic porphyrin **3** most likely targets external structures; meanwhile, compound **4** will damage internal biomolecules. The mechanisms of cellular death after PDI treatment with tri- and tetra-cationic porphyrins at biomolecular level have been investigated [38]. The tetra-cationic porphyrin showed a higher photodamage of plasmid and genomic DNA than the tri-cationic molecule. On the contrary, lipids in the cell membrane are an important target for tri-cationic porphyrins [39,42]. However, PSs are not limited to a single target such as most antibiotics; therefore, structural and catalytic proteins should be considered as key PDI targets [43,44].

To further corroborate that the internalization of **4** is carried out through the self-promoted uptake pathway a competitive binding experiment was carried out. This pathway involves the interaction of the cationic compounds at divalent cation binding sites, which promotes the internalization of the molecules. Therefore, the presence of excess divalent cations, such as Mg^+2^, may cause a competitive inhibition of the uptake of the cationic compounds [13,41]. Our results showed that increasing the concentrations of Mg^+2^ caused a reduction in the amount of **4** internalized by *E. coli* (Figure 7). Incubating cells in the presence of 50 mM Mg^+2^ reduced the uptake of the tetra-cationic porphyrin **4** by 35% (*p* < 0.05). This proves that the addition of excess Mg^+2^ prevents alterations in the outer membrane permeability functions of cells treated with **4**. Durantini et al. reported a similar effect with both Ca^+2^ and Mg^+2^ confirming that the uptake of **4** is mediated by electrostatic interactions and self-promoted uptake pathway [45]. On the other hand, incubating cells with 50 mM Mg^+2^ reduced the uptake of **3** by less than 10%. Therefore, these results confirm that the difference in the capacity of **3** and **4** to permeate the bacterial membrane is a factor that can explain the observed differences in the PDI performance of these cationic porphyrins.

## 4. Materials and Methods: Synthesis and Structural Characterization of Porphyrin Derivatives

### 4.1. Synthesis of 5-(4′-N,N,N-Trimethylammoniumphenyl)-10,15,20-Triphenylporphyrin Iodide (1)

The synthesis of porphyrin **1** was accomplished by alkylating commercially available 5-(4′-aminophenyl)-10,15,20-triphenylporphyrin [28]. A mixture of 5-(4′-aminophenyl)-10,15,20-triphenylporphyrin (45 mg, 0.0714 mmol) and methyl iodide (CH_3_I) (4 mL, 64.2 mmol) in anhydrous N,N-dimethylformamide (DMF) (5 mL) was heated to 45 °C and stirred for 24 h under nitrogen gas. Dichloromethane (CH_2_Cl_2_) (50 mL) was added to the reaction mixture, and the solution was washed with water. The aqueous and organic layers were separated using a separatory funnel. The organic layer was collected. The solvent was removed using rotary evaporation to dryness. A minimal amount of CH_2_Cl_2_ was added followed by an excess of diethyl ether. The desired porphyrin precipitated down and was collected by vacuum filtration. Yield: 76% wt. ^1^H NMR (300 MHz, CDCl_3_, ppm): δ 8.88–8.90 (2H, d, β-pyrrole), 8.84 (4H, s, β-pyrrole), 8.79–8.81 (2H, d, β-pyrrole), 8.50–8.53 (2H, d, *o*-N+Ph), 8.41–8.44 (2H, d, *m*-N+Ph), 8.21–8.24 (6H, d, o-Por), 7.83–7.86 (9H, m, *m*/*p*-Por), 3.92 (9H, s, CH_3_), −2.92 (2H, s, pyrrole-H). FTIR (solid, cm^−1^): 3445 (N–H stretch), 2852-3135 (C–H stretch), 1595 (C=C bend), 1474 (C–H bend), 965 (C–N stretch). ESI-MS (m/z): [M–I]^+^ = 672.20; Calculated for [M–I]^+^ = 672.31.

### 4.2. Synthesis of the Mixture cis-5,10-bis(4′-N,N,N-Trimethylammoniumphenyl)-15,20-Diphenylporphyrin Iodide and trans-5,15-bis(4′-N,N,N-trimethylammoniumphenyl)-10,20-Diphenylporphyrin Iodide (2)

#### 4.2.1. Synthesis of the Mixture cis-5,10-bis(4′-Nitrophenyl)-15,20-Diphenylporphyrin and trans-5,15-bis(4′-Nitrophenyl)-10,20-Diphenylporphyrin

TPP (127.6 mg, 0.207 mmol) was dissolved in CH_2_Cl_2_ (30 mL) and flushed with N_2_ at room temperature. NO_2_BF_4_ (500 µL, 0.250 mmol; 0.5 M in sulfolane) was added dropwise to the reaction mixture and stirred for 60 min. The nitration of TPP was monitored by thin layer chromatography (TLC). Four aliquots of NO_2_BF_4_ (0.625 mL) were added dropwise every 60 min for a total of 2.5 mL of NO_2_BF_4_ solution. After the final addition, TLC analysis showed a small amount of unreacted TPP and other red spots representing the mono- and di-nitrated porphyrins. To produce more di-nitrated porphyrin, the reaction was stirred under N_2_ for an additional 24 h. Next, the reaction mixture was extracted with water. The organic layer was collected, and the volatile removed under vacuum. To remove sulfolane, a minimal amount of acetone (<5 mL) was added, followed by cold water (50 mL) which caused the porphyrin mixture to precipitate. The porphyrin mixture was collected by vacuum filtration. MALDI-TOF analysis of the mixture showed that the reaction produced a mixture of TPP and mono- and di- nitrated porphyrins. Separation of the mixture was performed using silica chromatography and eluting with CH_2_Cl_2_: Hexanes (1:1). The desired di-nitrated porphyrin was collected as the third red band. Yield: 67% wt. ^1^H NMR (300 MHz, CDCl_3_, ppm): δ 8.52–8.66 (8H, m, β-pyrrole), 8.34–8.37 (4H, d, J = 8.6 Hz, *o*-Ph-NO_2_), 8.10–8.13 (4H, d, J = 8.6 Hz, m- Ph-NO_2_), 7.93–7.95 (4H, d, *o*-Ph-H), 7.52–7.59 (6H, m, *m*/*p*-Ph-H), −3.07 (2H, s, pyrrole-NH). FTIR (solid, cm^−1^): 3316 (N–H stretch), 2851–3102 (C–H stretch), 1594 (Aromatic), 1342, 1513 (NO_2_ stretch), 964 (C–N stretch). MALDI-TOF (m/z): [M]^+^ = 704.50. Calculated for [M]^+^ = 704.22.

#### 4.2.2. Synthesis of Mixture cis-5,10-bis(4′-Aminophenyl)-15,20-Diphenylporphyrin and trans-5,15-bis(4′-Aminophenyl)-10,20-Diphenylporphyrin

5,10-(4′-nitrophenyl)-15,20-triphenylporphyrin and 5,15-(4′-nitrophenyl)-10,20-triphenyl porphyrin mixture (97.4 mg, 0.138 mmol) was dissolved in 37% HCl (42.5 mL) and sonicated to assist dissolution. SnCl_2_∙H_2_O (1.35 g, 5.96 mmol) was added to this solution, and the reaction mixture was heated to 65 °C. The reaction was stirred for 24 h at 65 °C. D.I. H_2_O (50 mL) was added to dilute the solution. Then 0.1 M NH_4_OH was added until pH = 8 was reached. The solution was washed with chloroform (100 mL). The aqueous and organic layers were separated using a separatory funnel. The organic layer was collected, and the solvent was removed using rotary evaporation to dryness. Silica chromatography and chloroform were used to obtain the products. The desired di-amine porphyrin mixture eluted as the second band. Yield: 87% wt. ^1^H NMR (300 MHz, CDCl_3_, ppm): δ 8.80–8.92 (8H, m, β-pyrrole), 8.19–8.22 (4H, d, J = 8.7 Hz, *o*-Ph-H), 7.96–7.99 (4H, d, J = 8.7 Hz, *m*-Ph-NH_2_), 7.72–7.74 (6H, m, *m*/*p*-Ph-H), 7.01–7.04 (4H, d, *o*-Ph-NH_2_), −2.74 (2H, s, pyrrole-NH). FTIR (solid, cm^−1^): 3451, 3362, 3321 (N–H stretch), 2851–3026 (C–H stretch), 1468 (C–H bend), 1616 (aromatic). MALDI-TOF (m/z): [M]^+^ = 644.61; Calculated for [M]^+^ = 644.27.

#### 4.2.3. Synthesis of Mixture cis-5,10-bis(4′-N,N,N-Trimethylammoniumphenyl)-15,20-Diphenylporphyrin iodide and trans-5,15-bis(4′-N,N,N-Trimethylammoniumphenyl)-10,20-Diphenylporphyrin iodide (2)

5,10-(4′-aminophenyl)-15,20-triphenylporphyrin and 5,15-(4′-aminophenyl)-10,20-triphenylporphyrin mixture (26.5 mg, 0.0411 mmol) was dissolved in anhydrous DMF (5 mL) and flushed with N_2_ at room temperature. CH_3_I (4 mL, 64.25 mmol) was added and the reaction stirred for 24 h. After 24 h, CH_2_Cl_2_ (25 mL) and water (25 mL) were added to the reaction mixture. The aqueous and organic layers were separated using a separatory funnel. The desired porphyrin product and starting porphyrin resided in the organic layer. Therefore, the organic layer was collected, and the solvent was removed using rotary evaporation to dryness. CH_2_Cl_2_ (1 mL) was added back to the round bottom flask and an excess of diethyl ether was added which caused the alkylated porphyrin to precipitate down. The precipitate was collected by vacuum filtration. Yield: 75% wt. ^1^H NMR (300 MHz, DMSO-*d*_6_, ppm): δ 8.77–8.87 (8H, m, β-pyrrole), 8.47–8.50 (4H, d, J = 9.0 Hz, *o*-N+Ph), 8.41–8.44 (4H, d, J = 9.0 Hz, *m*-N+Ph), 8.19–8.22 (4H, d, *o*-Ph-H), 7.83–7.85 (6H, m, *m*/*p*-Ph-H), −2.95 (2H, s, pyrrole-NH), 3.91 (18H, s, CH_3_). FTIR (solid, cm^−1^): 3317, 3422 (N–H stretch), 2851–2988 (C–H stretch), 1596 (aromatic). ESI-MS (m/z): [M–2(I^−^)-2H]^+^ = 364.49; Calculated for [M–2(I^−^)-2H]^+^ = 364.19.

### 4.3. Synthesis of Mixture 5,10,15-tris(4′-N,N,N-Trimethylammoniumphenyl)-20-Phenylporphyrin Iodide (3)

#### 4.3.1. Synthesis of Mixture 5,10,15-tris(4′-Nitrophenyl)-20-Phenylporphyrin Iodide

TPP (120.1 mg, 0.195 mmol) was dissolved in CH_2_Cl_2_ (30 mL) and flushed with N_2_ at room temperature. One aliquot of NO_2_BF_4_ (500 µL, 0.250 mmol; 0.5 M in sulfolane) was added dropwise to the reaction vessel. The reaction mixture stirred at room temperature for 60 min. Additional aliquots of NO_2_BF_4_ (500 µL, 0.250 mmol; 0.5 M in sulfolane) was added every 60 min until TLC analysis indicated a complete consumption of TPP. A total of ten aliquot of NO_2_BF_4_ were added (5.0 mL, 2.5 mmol). After the final addition, the reaction stirred under N_2_ for an additional 24 h. The reaction mixture was extracted with water. The organic layer was collected, and the volatile removed under vacuum. To remove sulfolane, a minimal amount of acetone (<5 mL) was added, followed by cold water (25 mL) which caused the porphyrin mixture to precipitate. The porphyrin mixture was collected by vacuum filtration. MALDI-TOF analysis of the mixture showed that the reaction produced a mixture of mono-, di-, and tri-nitrated porphyrins. Separation of the mixture was performed using silica chromatography and eluting with CH_2_Cl_2_:Hexanes (1:1). The desired tri-nitrated porphyrin was collected as the third red band. Yield: 44% wt. ^1^H NMR (300 MHz, CDCl_3_, ppm): δ 8.83–8.84 (2H, d, β-pyrrole), 8.73 (4H, s, β-pyrrole), 8.68–8.70 (2H, d, β-pyrrole), 8.56–8.59 (6H, d, J = 8.3 Hz, *o*-Ph-NO_2_), 8.31–8.33 (6H, d, J = 8.3 Hz, *m*-Ph-NO_2_), 8.10–8.12 (2H, d, *o*-Ph-H), 7.69–7.71 (3H, m, *m*/*p*-Ph-H), −2.91 (2H, s, pyrrole-NH). FTIR (solid, cm^−1^): 3315 (N–H stretch), 2857–3046 (C–H stretch), 1593 (aromatic), 1341–1512 (NO_2_). MALDI-TOF (m/z): [M+1]^+^ = 750.65. Calculated for [M+1]^+^ = 750.20.

#### 4.3.2. Synthesis of Mixture 5,10,15-tris(4′-Aminophenyl)-20-Phenylporphyrin Iodide

5,10,15-tris(4′-nitrophenyl)-20-phenylporphyrin (34.1 mg, 0.0455 mmol) was dissolved in 37% HCl (25 mL) and sonicated to assist dissolution. SnCl_2_∙H_2_O (0.67 g, 3.009 mmol) was added to this solution, and the reaction mixture was heated to 65 °C. The reaction was stirred for 24 h. D.I. H_2_O (25 mL) was added to dilute the solution. Then 0.1 M NH_4_OH to diluted solution until a pH = 8 was reached. The solution was washed with chloroform (50 mL). The aqueous and organic layers were separated using a separatory funnel. The organic layer was collected, and the solvent was removed using rotary evaporation to dryness. Silica chromatography was used to purify the product. The mixture was eluted with chloroform and methanol (1%) and the second band was collected giving the desired tri-amine porphyrin. Yield: 40% wt. ^1^H NMR (300 MHz, CDCl_3_, ppm): δ 8.92–8.93 (6H, m, β-pyrrole), 8.81–8.83 (2H, d, β-pyrrole), 8.22 (2H, d, *o*-Ph-H), 7.95–7.98 (6H, d, J = 8.2 Hz, *m*-Ph-NH_2_), 7.73 (3H, m, *m*/*p*-Ph-H), 6.97–6.99 (6H, d, J = 8.2 Hz, *o*-Ph-NH_2_), −2.68 (2H, s, pyrrole-NH). FTIR (solid, cm^−1^): 3316 (N–H stretch), 2853–2922 (C–H stretch), 1598 (aromatic), 1473 (C–H stretch), 1035 (C–N stretch). MALDI-TOF (m/z): [M–1]^+^ = 658.51, [M]^+^ = 659.58. Calculated for [M]^+^ = 659.28.

#### 4.3.3. Synthesis of Mixture 5,10,15-tris(4′-N,N,N-Trimethylammoniumphenyl)-20-Phenylporphyrin Iodide (3)

5,10,15-tris(4′-aminophenyl)-20-phenylporphyrin (39.1 mg, 0.059 mmol) was dissolved in anhydrous DMF (5 mL) and flushed with N_2_ at room temperature. CH_3_I (6 mL, 96.378 mmol) was added and the reaction stirred for 24 h at 45 °C. After 24 h, an excess of acetone (25 mL) was added which caused the alkylated porphyrin to precipitate down. The precipitate was collected by vacuum filtration. Yield: 28% wt. ^1^H NMR (300 MHz, DMSO-*d*_6_, ppm): δ 8.85–8.91 (6H, m, β-pyrrole), 8.79–8.80 (2H, d, β-pyrrole), 8.49–8.52 (6H, d, J = 9.4 Hz, *o*-Ph-N+), 8.42–8.45 (6H, d, J = 9.4 Hz, *m*-Ph-N+), 8.20–8.23 (2H, d, *o*-Ph-H), 7.78–7.87 (3H, m, *m*/*p*-Ph-H), 3.93 (27H, s, CH_3_), −2.96 (2H, s, pyrrole-NH). FTIR (solid, cm^−1^): 3397, 3316 (N–H stretch), 2849–3005 (C–H stretch), 1597 (aromatic). ESI-MS (m/z): [M–3(I^−^)]^+^ = 262.58; Calculated for [M–3(I^−^)]^+^ = 262.81.

#### 4.3.4. Synthesis of Mixture 5,10,15-tetra(4′-N,N,N-Trimethylammoniumphenyl)-20-Phenylporphyrin Iodide (4)

A mixture of 5,10,15,20-tetrakis(4′-aminophenyl) porphyrin (49.7 mg, 0.0737 mmol) and CH_3_I (4 mL, 64.25 mmol) in anhydrous DMF (10 mL) was heated to 45 °C and stirred for 24 h under N_2_. After 24 h, excess acetone (25 mL) was added to precipitate down the product. The precipitate was collected by vacuum filtration. Yield 68% wt. ^1^H NMR (500 MHz, DMSO-*d*_6_, ppm): δ 8.89 (8H, s, β-pyrrole), 8.49–8.51 (8H, d, J = 9.6 Hz, *m*-N+), 8.46–8.48 (8H, d, J = 9.6 Hz, *o*-N+), 3.96 (36H, s, CH_3_), −2.94 (2H, s, pyrrole-H). FTIR (solid, cm^−1^): 3420 (N–H stretch), 2752–3019 (C–H stretch), 1460 (C–H bend), 1577 (Aromatic). ESI-MS (m/z): [M–4(I^−^)]^+^ = 211.80. Calculated for [M–4(I^−^)]^+^ = 211.63.

### 4.4. Stock Solutions

Stock solutions (1 mM) of each porphyrin were prepared in DMSO and DMF. For biological assays, the stock solutions of photosensitizers prepared in DMSO were diluted to the final concentrations in phosphate buffer solution (PBS, 1X, pH 7.4) of 1% DMSO.

### 4.5. Photophysical Characterization of Cationic Porphyrins

#### 4.5.1. Absorbance and Emission Studies

Absorption and emission measurements for the cationic porphyrins were collected on solutions of 5 µM in DMSO. The fluorescence quantum yields for air-saturated solutions (Φ_F_) in DMSO were determined using the comparative method, as shown in Equation (2). TPP with a quantum yield of 0.12 benzene was used as the reference [14]. The porphyrin concentration ranged from 3 to 15 µM in DMSO. The excitation wavelength was 520 nm and the excitation and emission slit width were 2 nm. The integrated area was measured using Origin (fluorescence software), and the slope of the best fit line was determined using GraphPad Prism version 7.03 for Windows, GraphPad Software, La Jolla CA, USA, www.graphpad.com.

(2) $$\Phi_{F,\mathrm{Sample}}\;=\;\Phi_{F,\mathrm{Reference}}\;\times \;\frac{m_{\mathrm{Sample}}}{m_{\mathrm{Reference}}}\;\times \;\frac{n_{\;\mathrm{Sample}}^{2}}{n_{\;\mathrm{Reference}}^{2}}$$

#### 4.5.2. Singlet Oxygen Quantum Yield Studies

The quantum yields of singlet oxygen were determined through the absorbance decay of DMA in DMF using the comparative method, as shown in Equation (1). Solutions containing DMA (50 µM) and the PS (~5 µM; OD ≈ 0.1 at irradiation wavelength) were prepared in DMF saturated with oxygen. After preparation, the solutions were protected from light. A volume of 2 mL of the solution was filled in a quartz cuvette (1 cm × 1 cm), set into a fluorometer (xenon lamp, Shimadzu RF5301 PC) and irradiated at 515 nm. The irradiation period, controlled by a shutter, was maintained for time intervals (0–600 s). The decay of DMA was monitored at 379 nm. A reference spectrum of the PS in DMF was taken before each experiment and subtracted from the final data. The experiments were performed at least two times for each PS. The experimental design was confirmed by comparison with two references, TPP (Φ_Δ_ = 0.62) [21] and **5** (Φ_Δ_ = 0.54) [29] in DMF. The slope of the time-dependent decay of DMA absorbance plot was determined using linear regression analysis in GraphPad Prism version 7.03 for Windows, GraphPad Software, La Jolla, CA, USA, www.graphpad.com.

### 4.6. In Vitro Characterization of Cationic Porphyrins with E. coli

#### 4.6.1. Bacterial Growth Conditions

*E. coli* (427) was inoculated in liquid Luria Broth (LB) containing 50 µg/mL kanamycin and grew at 37 °C for approximately 6–8 h until the optical density at 600 nm (OD_600nm_) reached approximately 0.8 (a.u.). Cell suspension solutions were prepared using PBS (1X, pH = 7.4). Bacterial cells were harvested through centrifuging (5 × 10^3^× *g* for 10 min, 4 °C) and washed with PBS. After discarding the supernatant, the remaining bacterial cell pellet was resuspended in PBS, 1% DMSO solution (1.0 mL) to an OD_600nm_ ~0.6–0.8.

#### 4.6.2. Irradiation Conditions

The effect of the cationic porphyrin derivatives was evaluated by exposing bacterial suspensions to white light (400–700 nm) with a distance of 10 cm from the surface of the cell culture to the light source, and at an irradiance of 44 J cm^−2^ for 20 min without stirring. The light power density was measured with OPHIR VEGA power/energy meter.

#### 4.6.3. Photodynamic Inactivation Assays

A bacterial suspension (OD_600nm_ ~0.6–0.8; 3–4 10^8^ CFU/mL) in PBS (1% DMSO) solution (1.0 mL) was incubated at 37 °C for 30 min in the dark in the presence of each porphyrin (concentration = 1 nM to 10 µM). After 30 min, an aliquot of 100 µL representing the “dark toxicity” was removed for each experiment and stored in a 1.5 mL Eppendorf centrifuge tube. The original cell suspension was centrifuged (5 × 10^3^ × *g*, 10 min, 4 °C) to remove unbound PS. The obtained bacterial cell pellets were resuspended in PBS (1% DMSO) solution (900 µL) and exposed to white light (44 J cm^−2^) for 20 min. After white light exposure, another aliquot of 100 µL representing the “light toxicity” sample was removed and stored in a new 1.5 mL Eppendorf centrifuge tube.

The survival percentage was determined by counting the colony-forming units (CFU). The control, dark toxicity and light toxicity solutions were serially diluted 10^6^-fold with autoclaved distilled water. The drop-plate method was used to plate diluted samples onto agar plates. Four aliquots of 10 µL portions of the diluted bacterial suspensions were pipetted onto solid LB agar plates which contained 50 µg/mL Kanamycin. After incubating for 24 h at 37 °C bacterial colonies were formed. The dilution containing 3–30 colonies was counted. The CFU per mL for each sample was determined by dividing the average CFU by the volume plated in mL (0.010 mL) and the dilution factor. The cell survival percentage of the dark and light samples were calculated as a percent of the control using Equation (3).

(3) $${\mathrm{Survival}\;\%}_{\mathrm{Dark}/\mathrm{Light}\;\mathrm{Toxicity}}\;=\;\frac{{\mathrm{CFU}}_{\mathrm{Dark}/\mathrm{Light}\;\mathrm{Toxicity}}}{{\mathrm{CFU}}_{\mathrm{Control}}}\;\times \;100\%$$

### 4.7. Live-Cell Fluorescence Microscopy

To visualize the interaction between bacterial cells and cationic porphyrins, fluorescence live-cell deconvolution microscopy was used. Bacterial suspensions (OD_600nm_ ~0.6–0.8; 3–4 10^8^ CFU/mL) were incubated with each porphyrin (concentration = 1 µM) at 37 °C for 30 min in the dark. After discarding the unbound PS by centrifugation (5 × 10^3^× *g*, 10 min, 4 °C), the cell pellet and tightly-bound porphyrin was resuspended in PBS (1X, pH= 7.4, 500 µL). Aliquots of 40 µL cell suspensions were pipetted to glass slides containing agarose pads (0.7%).

Samples were excited with 390 nm and emission captured at 679 nm using Delta Vision’s Standard DAPI/FITC/TRITC/Cy5 filter set. Z-stack images were collected at 0.2 µm z-increments on a DeltaVision Elite Workstation (GE-Helathcare) based on an inverted microscope (1X-70; Olympus) using a 100×/1.4 NA oil immersion lens and Nomarski optics. Images were captured at room temperature with a 12-bit CCD camera (Cool Snap HQ; Photometric) and deconvoluted using the interactive-constrained algorithm and the measured point spread function. All image analyses were performed using ImageJ v1.51n (National Institute of Health).

### 4.8. Competitive Binding

To confirm that the cellular internalization of porphyrin **4** into bacterial cells occurred via the self-promoted uptake pathway, the uptake of compounds **4** and **3** into *E. coli* cells was studies by incubating the tetracationic porphyrin in bacterial culture solutions pre-exposed to increasing concentrations of Mg^+2^, source MgCl_2_. A stock solution of MgCl_2_ (1 M in autoclaved DI water) was prepared. Next, different volumes of MgCl_2_ (1M in autoclaved DI water) were added to bacterial suspensions (OD_600nm_ ~0.6–0.8) in PBS solution (1.0 mL) to obtain final concentrations of 0, 10, 25, and 50 mM MgCl_2_. The bacterial solutions were incubated at 37 °C for 30 min. The bacterial solutions were centrifuged (5000× *g* for 10 min, 4 °C) to remove unbound Mg^+2^. The obtained bacterial pellets were dispersed in a pre-made PS solution ([PS] = 1 µM; 1 mL PBS). Then the PS/bacterial mixture was incubated at 37 °C and protected from light for 30 min. After incubation, unbound PS was removed from the suspension by centrifuging (5000× *g* for 10 min, 4 °C). To extract the cell-bound PS from the bacterial cells, the obtained pellet was dispersed in 2% sodium dodecyl sulfate (SDS) at room temperature for 14 h. The concentration of PS in solution was analyzed by fluorescence. The samples were excited at 415 nm and the emission of the PS was monitored at the fluorescence maximum (**3**, 652 nm and **4**, 652 nm). The PS concentration was found by interpolation with a calibration plot constructed with known concentrations of each PS in 2% SDS (Figure S2). Three experiment replicates were performed.

### 4.9. Statistical Analysis

Statistical analyses were performed by using GraphPad Prism (v7.03 for Windows, La Jolla, CA, USA). The significance of charge number and porphyrin concentration on bacterial inactivation was assessed by one-way univariate analysis of variance (ANOVA) model with the Tukey’s post hoc test. A value of *p* < 0.05 was considered as statistically significant.

## 5. Conclusions

The results in this study confirmed that the number of cationic groups on porphyrins has a major impact on the PDI of *E. coli*. The dose-response graph showed that the PDI efficiency of the porphyrins against *E. coli* was directly correlated with the number of positive charges following the order: **5** < **1** < **2** < **3**; nevertheless, this trend is not followed by compounds **4** and **6**. Hill coefficients obtained from the dose-response data indicate that compounds **1**–**3** may follow a different mechanism of inactivation than compounds **4** and **6**. Fluorescent live-cell microscopy revealed that indeed the interaction of compounds **1**–**3** with *E. coli* is completely different than the one for compounds **4** and **6**. Under the conditions used for our experiments, compounds **1**–**3** are localized in the cell membrane; however, compounds **4** and **6** have already been internalized by *E coli* cells. The contrast in the cellular localization of the cationic porphyrin derivatives leads to different molecular targets for the PDI. These results shed light to explain the difference in PDI efficacy between cationic porphyrin derivatives against *E. coli*. Nevertheless, the mechanisms of cellular death after PDI treatment with these cationic porphyrins derivatives at biomolecular level needs to be further investigated.

## Figures and Tables

**Table 1:** Photophysical parameters for cationic porphyrins including extinction coefficient values (ε), fluorescence (Φ_F_) and ^1^O_2_ (Φ_Δ_) quantum yields.

Porphyrin	λ_Soret_ (nm) [ε × 10^3^ (M^−1^ cm^−1^)]	λ_Emission_ (nm) ^a^	Φ_F_ (520 nm) ^b^ (n = 3)	Φ_Δ_^c^ (n = 3)
**1**	418 [355 ± 22]	649, 716	0.11 ± 0.01	0.65 ± 0.04
**2**	418 [285 ± 19]	649, 715	0.10 ± 0.01	0.61 ± 0.03
**3**	418 [248 ± 30]	648, 715	0.12 ± 0.01	0.61 ± 0.06
**4**	418 [324 ± 38]	647, 713	0.11 ± 0.01	0.65 ± 0.04

^a^ λ_ex_ = 520 nm. ^b^ Fluorescence quantum yields in DMSO were calculated based on the fluorescence spectra using TPP (Benzene) as a standard (Φ_F_ = 0.12) [14]; λ_ex_ = 520 nm. ^c 1^O_2_ quantum yields in DMF were calculated using TPP (DMF) as a standard (Φ_Δ_ = 0.62) [21]; λ_Irradiation_ = 515 nm.

**Table 2:** Analysis of PDI efficiency using the EC_50_ and EC_99.99_ parameters. Values for EC_50_ were determined by best fit analysis of a dose response curve on data from at least three independent experiments, with additional experiments using concentrations at or near the initial EC_50_ value for refinement. Hill coefficients were determined by analysis of log plots and adjusted incrementally for the best fit to the higher concentrations that did not result in complete inactivation. EC_99.99_ values were calculated from the EC_50_ and Hill coefficient with error propagation from the EC_50_ measurement. Error in the EC_50_ represents one standard of deviation from the fit mean value. All data for these measurements is provided in Figure S1.

Porphyrin	EC_50_ (nM)	EC_99.99_ (nM)	Hill
**1**	146 ± 13	5810 ± 520	2.5
**2**	10.7 ± 1.4	425 ± 56	2.5
**3**	1.3 ± 0.3	51 ± 11	2.5
**4**	11.4 ± 0.6	52.9 ± 2.8	6.0
**6**	29.0 ± 3.2	290 ± 32	4.0

## Supplementary Materials

Supplementary materials can be found at http://www.mdpi.com/1422-0067/20/1/134/s1.

## Abbreviations

PDI	Photodynamic inactivation
PS	Photosensitizer
^1^O_2_	Singlet oxygen
ROS	Reactive oxygen species
LPS	Lipopolysaccharides
*E. coli*	Escherichia coli
TPP	Tetraphenyl porphyrin
DMSO	Dimethyl sulfoxide
ISC	Intersystem crossing
ε	Extinction coefficient values
Φ_F_	Fluorescence quantum yield
Φ_Δ_	Singlet oxygen quantum yield
DMF	Dimethyl formamide
DMA	9,10-dimethylantracene
CFU	Colony-forming units
EC	Effective concentration
SDS	Sodium dodecyl sulfate
