# Supplementary material for: Influence of Cationic meso-Substituted Porphyrins on the Antimicrobial Photodynamic Efficacy and Cell Membrane Interaction in Escherichia coli
Source: Int J Mol Sci. 2019 Jan 1;20(1):134. doi: 10.3390/ijms20010134 (PMC6337135; doi:10.3390/ijms20010134)
Supplement: Supplementary file 1 [file ijms-20-00134-s001.pdf]

Supplementary Material

Influence of cationic *meso*-substituted porphyrins on the photodynamic inactivation and cell membrane interaction with *Escherichia coli*

Alexandra N. Hurst[1,3,4], Beth Scarbrough[1,3,4], Roa Saleh[1], Jessica Hovey[1], Farideh Ari[1], Shreya Goyal[2,3,], Richard J. Chi[2,3], Jerry M. Troutman[1,3,4,*]; Juan L. Vivero-Escoto[1,3,4,*]

[1] *Department of Chemistry, The University of North Carolina at Charlotte, Charlotte NC 28223, USA*
[2] *Department of Biological Sciences, The University of North Carolina at Charlotte, Charlotte NC 28223, USA*
[3] *The Center for Biomedical Engineering and Science, The University of North Carolina at Charlotte, Charlotte NC 28223, USA*
[4] *Nanoscale Science Program, Department of Chemistry, The University of North Carolina at Charlotte, Charlotte NC 28223, USA*

Materials and Methods

All commercial chemicals and solvents were of reagent grade or higher and were used as received. Tetraphenyl porphyrin (TPP) was purchased from TCI. 5,10,15,20-tetrakis(3-hydroxyphenyl)porphyrin (**5**) and 5,10,15,20-tetrakis(N-methylpyridinium)porphyrin iodide (**6**) were purchased from PorphyChem. All experiments with moisture/air-sensitivity were performed in anhydrous solvents under a nitrogen atmosphere. Column chromatography was performed using silica G60 (70-230 mesh). IR spectra were recorded on a Perkin-Elmer 100 IR spectrophotometer. ^{1}H NMR spectra were recorded on a 300 MHz or 500 MHz JEOL NMR spectrometer and are referenced with $CDCl_3$ and DMSO-d_6 solvents. Mass spectra were obtained using a Voyager Biospectrometry Laser MALDI-TOF spectrometer or Thermal Scientific MSQ Plus ESI spectrometer. UV-Vis spectra were recorded on a Cary 300 UV-visible spectrophotometer. Steady-state fluorescence emission was measured on a Jobin Yvon Fluorolog 3. High resolution fluorescence microscopy z-stacking images were collected using a DeltaVision Elite Workstation based on an inverted microscope (1X-70; Olympus) equipped with a 100x, 1.4 NA oil immersion lens.

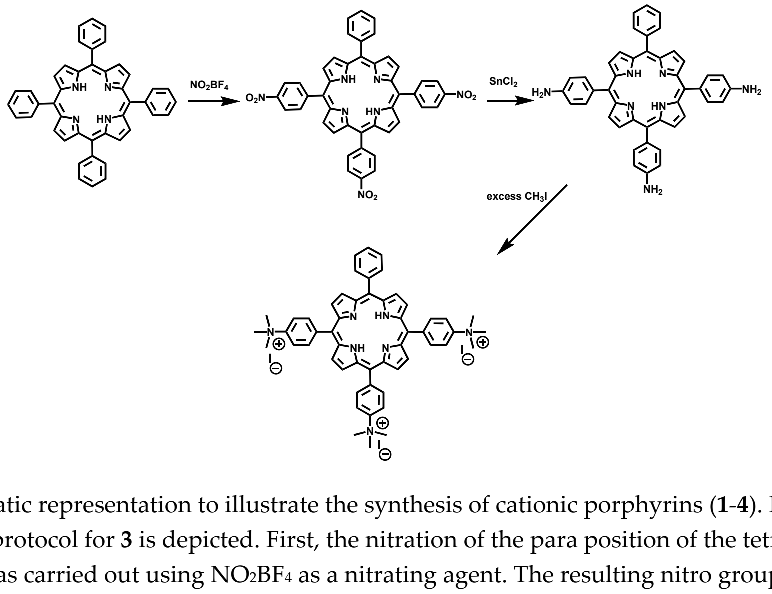

Scheme S1. Schematic representation to illustrate the synthesis of cationic porphyrins (**1**-**4**). For simplicity, only the synthetic protocol for **3** is depicted. First, the nitration of the para position of the tetraphenyl porphyrin (TPP) was carried out using NO_2BF_4 as a nitrating agent. The resulting nitro groups were then reduced with $SnCl_2$ in acidic medium. In the final step, the amino phenyl porphyrin derivatives were alkylated using a large excess of methyl iodide.Porphyrin **2** was synthesized following a similar approach. Cationic porphyrins **1** and **4** were synthesized by methylation with CH_3I from the corresponding amino derivatives.

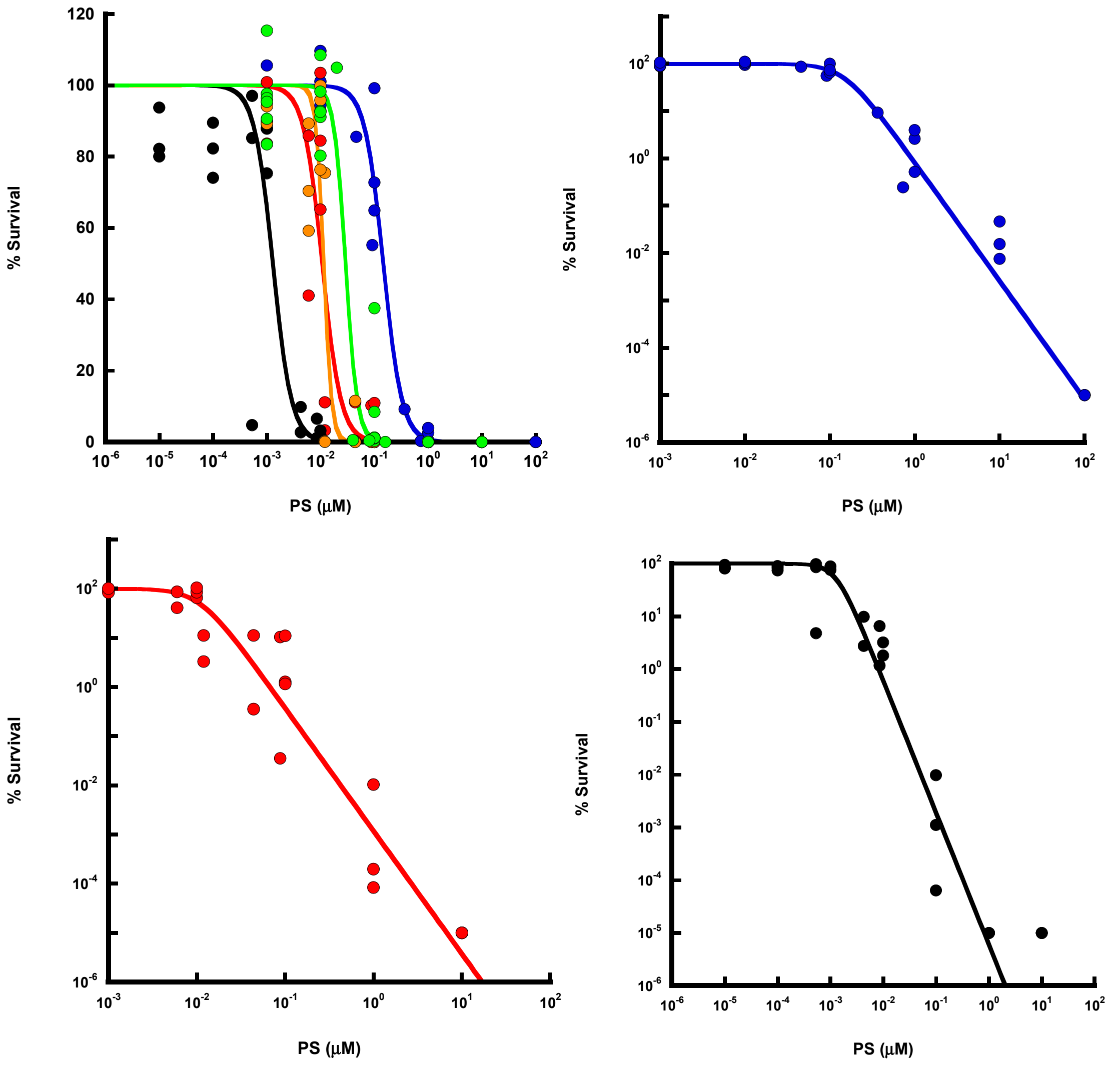

% Survival
PS (μM)
% Survival
PS (μM)
% Survival
PS (μM)
% Survival
PS (μM)

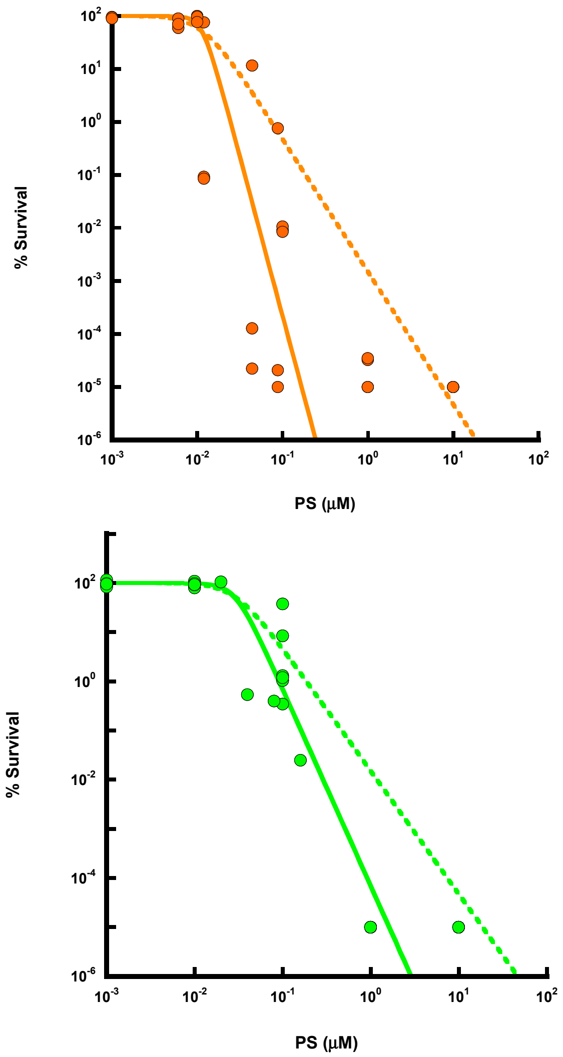

Figure S1. EC_{50} survival curve of **1** (blue), **2** (red), **3** (black), **4** (orange) and **6** (green) against E. coli. Values are reported as a percent survival relative to DMSO treated controls. Data obtained for a minimum of three independent experiments with fresh cultures. Plots depict the same data with a linear (top left) or log (all others) % survival-axis. Hill coefficients were assigned as shown in Table 2. Hill coefficients were weighted to fit the higher concentration data that did not lead to complete inactivation. Zero is given a 10^{-5} % survival, which is the detection limit of the assay. All replicate points at a given concentration were from independent experiments. Fitting statistics for each compound were as follows: **1**: R=0.9801, **2**: R=0.8742, **3**: R=0.8777, **4**: R=0.9002, **6**: R=0.9749. The lower two plots are of data with compounds **4** and **6** using the Hill coefficient of 2.5 (dotted line) versus the coefficient used here to determine EC_{50} (solid line).

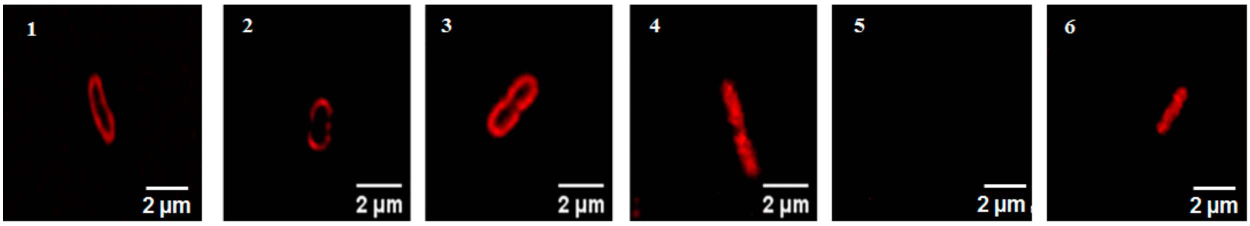

Figure S2. Fluorescence micrographs depicting the interaction of cationic porphyrins **1-6** with *E. coli.* [PS] = 1 µM; Incubation time: 30 min.

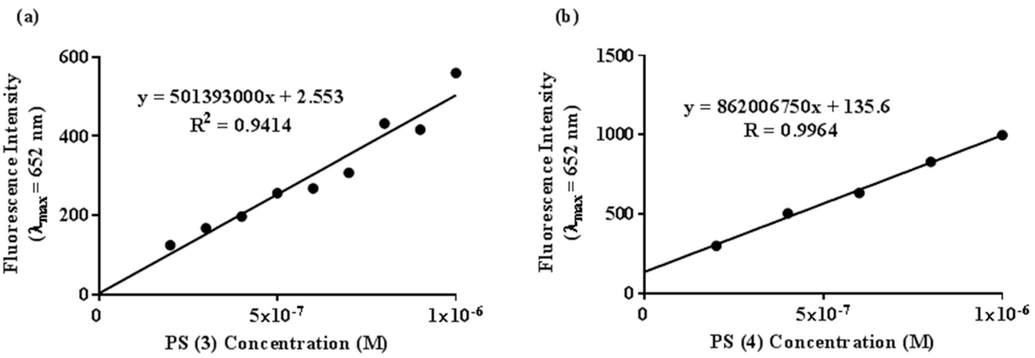

Figure S3. Calibration curves were constructed for **3** and **4** in 2% SDS.
